# Säuglings‑, Kinder- und Jugendlichen- (SKJ) Psychotherapien während der Covid-19 Pandemie: Ergebnisse einer Studie unter psychodynamischen Psychotherapeut*innen in Österreich

**DOI:** 10.1007/s00729-022-00213-9

**Published:** 2022-12-13

**Authors:** Brigitte Fiala-Baumann, Helga Ploner, Dominik Witzmann, Andrea Jesser

**Affiliations:** 1 Innsbruck, Österreich; 2 Wien, Österreich; 3 Wissenschaftliche Arbeitsgruppe, D.O.T. – Die offene Tür, Karl Landsteiner Privatuniversität für Gesundheitswissenschaften, Dr. Karl-Dorrek-Straße 30, 3500 Krems, Österreich

**Keywords:** Säuglingspsychotherapie, Kinderpsychotherapie, Jugendlichenpsychotherapie, Psychische Gesundheit von Kindern und Jugendlichen, Covid-19, Infant psychotherapy, Child psychotherapy, Adolescent psychotherapy, Child and Adolescent Mental health, Covid-19

## Abstract

**Fragestellung:**

Diese explorative Studie untersucht die Situation der Säuglings‑, Kinder- und Jugendlichen- (SKJ) Psychotherapien während der Covid-19 Pandemie in Österreich.

**Methodik:**

23 psychodynamisch arbeitende Psychotherapeut*innen nahmen an einer Online-Umfrage teil, die quantitative und qualitative Daten generierte. Offene Fragen zu subjektiv wahrgenommenen Veränderungen wurden mithilfe der qualitativen Inhaltsanalyse ausgewertet.

**Ergebnisse:**

Insgesamt sank die Anzahl an Psychotherapien insbesondere bei den Säuglings- und Kleinkind-Psychotherapien. Die Zahl der Kinder und Jugendlichen-Psychotherapien nahm zunächst ab, stieg nach dem ersten Lockdown aber an. Große Nachfrage wurde zuletzt vor allem bei Jugendlichenpsychotherapien wahrgenommen. Anpassungsleistungen an sich ändernde Bedingungen und Settings erforderten große Flexibilität der Psychotherapeut*innen, die mit Fortschreiten der Pandemie zunehmend an ihre Belastungsgrenzen kamen. Befragte beobachteten eine Zunahme an Symptomen sowie einen gestiegenen Bedarf an Eltern- und Vernetzungsarbeit mit Institutionen.

**Schlussfolgerungen:**

Tele-Psychotherapie kann vor allem mit jüngeren Kindern Präsenztherapie nicht ersetzen. Ergebnisse deuten auf eine Unterversorgung dieser Zielgruppe hin. Dies, sowie die beobachtete Zunahme an Symptomen bei älteren Kindern und Jugendlichen, ist *gesellschaftspolitisch und gesundheitsökonomisch ein großes Thema *und erfordert dringend einen weiteren Ausbau der Versorgungsnetzwerke.

## Abstract (en)

**Objective:**

This explorative study examines the situation of infant, child and adolescent psychotherapies during the Covid-19 pandemic in Austria.

**Method:**

23 psychodynamic psychotherapists participated in an online questionnaire that generated quantitative and qualitative data. Open questions on subjectively perceived changes were analysed using qualitative content analysis.

**Results:**

Overall, the number of psychotherapies decreased, especially for infant and toddler psychotherapies. The number of child and adolescent psychotherapies initially decreased but increased after the first lockdown. Great demand was recently perceived for adolescent psychotherapies. Adaptation to changing conditions and settings required great flexibility on the part of psychotherapists, who increasingly reached their limits as the pandemic progressed. Respondents observed an increase in symptoms as well as an increased need for work with parents and networking with institutions.

**Conclusions:**

Remote psychotherapy cannot replace in-person psychotherapy, especially with younger children. Results indicate a lack of treatment options for this target group. This, as well as the observed increase in symptoms in older children and adolescents, is a major issue from a socio-political and health economic point of view and urgently requires further expansion of psycho-social services and psychotherapeutic treatment options.

## Einleitung

Die Covid-19 Pandemie bestimmt seit mehr als zwei Jahren das gesellschaftliche Leben. Sie hat neben unmittelbaren Folgen für die körperliche Gesundheit durch eine Ansteckung mit dem Sars-Cov-2-Virus auch gravierende Auswirkungen auf die psychische Gesundheit – insbesondere bei jungen Menschen (Ma et al. 2021; Ravens-Sieberer et al. 2022). Studienergebnisse aus Österreich zeigen, dass schon Volksschüler*innen große Ängste in Bezug auf Corona aufweisen und ihnen der normale Alltag fehlt (Schabus und Eigl 2021). Über das psychische Wohlbefinden von Kindern gibt es aktuell noch keine Untersuchungen aus Österreich. Eine Umfrage unter Jugendlichen im Alter von 14 bis 20 Jahren findet bei über 50 % der jungen Menschen Symptome einer depressiven Erkrankung und bei über 45 % Symptome einer Angsterkrankung (Pieh et al. 2021). Im Zeitverlauf zwischen Februar und November 2021 bilden sich keine wesentlichen Veränderungen ab, was auf eine anhaltend hohe psychische Belastung der Jugendlichen schließen lässt. Gesellschaftlich ist das ein großes Problem, da die Generation der jetzt Kinder und Jugendlichen unter anderem die wirtschaftliche Zukunft darstellt. Es zeigt sich, dass belastete Jugendliche später zu seelisch belasteten Erwachsenen werden können (Fuchs et al. 2016). Ein Nichtbeachten der Belastungen dieser Gruppe kann zu hohen Kosten im Gesundheitssystem und im schlimmsten Fall zu späteren Ausfällen an Arbeitskräften führen.

### Psychosoziale Versorgungssituation

Die steigende Prävalenz psychischer Erkrankungen stellt eine Herausforderung für die psychosoziale Versorgungslandschaft dar. Aufgrund von Präventionsmaßnahmen war es zu Pandemiebeginn in vielen Bereichen erforderlich, Angebote der psychosozialen Versorgung zu adaptieren (Mädge et al. 2022). Der Zugang zu psychosozialen Unterstützungsangeboten über Telefon und Internet wurde wichtiger. Mit der zunehmenden Öffnung der Schulen wurde begonnen, niederschwellige Angebote auszubauen, die Kinder und Jugendliche in ihrem Lebensraum, z. B. in der Schule, zu erreichen versuchen. Als Beispiel kann hier das Projekt fit4SCHOOL (ÖBVP 2021) genannt werden. Ebenfalls wurden Betroffene über soziale Medien zu erreichen versucht. Mit dem Projekt „Gesund aus der Krise“ konnten außerdem 8000 (Kurzzeit)Psychotherapieplätze für Kinder und Jugendliche geschaffen werden (BÖP 2022). Trotz dieser Bemühungen und über 1000 Psychotherapeut*innen mit Weiterbildung in SKJ-Psychotherapie zeigt sich im Bereich der psychotherapeutischen Angebote nach wie vor eine Unterversorgung (Culen et al. 2020). Diese ist großteils auf eine fehlende Finanzierung zurückzuführen: wenige und teilweise schlecht bezahlte Kassenplätze führen dazu, dass viele Hilfesuchende gar keine Unterstützung finden – oder nur nach langen Wartezeiten (Mauritz 2019).

### SKJ Psychotherapie während der Pandemie

Während des ersten bundesweiten Lockdowns von 16. März bis 1. Mai 2020 schlossen viele Psychotherapeut*innen ihre Praxen für die Arbeit in Präsenz (Humer und Probst 2020; Probst et al. 2020). Da SKJ-Psychotherapie ein hohes Maß an Arbeit in Präsenz erfordert, war es hilfreich als klar wurde, dass Psychotherapie zu den Gesundheitsdienstleistungen gehört, die trotz Ausgangsbeschränkungen durchgeführt werden können. Aber auch die Arbeit unter erhöhten Sicherheitsauflagen stellte für die SKJ-Psychotherapie eine Herausforderung dar. Maskenpflicht und Abstandsregelungen erschwerten besonders die Arbeit mit Kleinkindern. Zudem wiesen Empfehlungen des Gesundheitsministeriums und der Berufsverbände darauf hin, wo möglich auf Tele-Psychotherapie umzusteigen.

Obwohl Forschungsergebnisse auf die Wirksamkeit internet- und telefonbasierter Interventionen für Kinder und Jugendliche hindeuten (Domhardt et al. 2018), ist die Studienlage zur psychotherapeutischen Versorgung von Säuglingen, Kindern und Jugendlichen während der Pandemie dünn. Telefon- und videobasierte Psychotherapie für Kinder scheint wenig Anwendung gefunden zu haben – Psychotherapien wurden schon bald wieder auf Präsenz-Therapien umgestellt (Hoffnung et al. 2021). Huscsava et al. (2020) berichten von guten Erfahrungen in der ambulanten Behandlung jugendlicher Patient*innen mit Hilfe von Videotelefonie. Andere Autor*innen streichen in Bezug auf die tele-psychotherapeutische Behandlung jugendlicher Patient*innen Vorteile wie den leichten Zugang, eine niedrige Hemmschwelle, Vertrautheit der Jugendlichen mit digitaler Kommunikation, reduziertes Stigma und die Ermächtigung der jungen Menschen durch vermehrte Mitgestaltungsmöglichkeiten hervor (Boydell et al. 2010; Hawke et al. 2021; Sweeney et al. 2019). Haslinger et al. (2021) beschreiben etwas differenzierter auch Einschränkungen der tele-psychotherapeutischen Arbeit mit Kindern und Jugendlichen, wie das Fehlen des persönlichen Kontakts und Einschränkungen hinsichtlich therapeutischer Techniken. Die Besonderheiten der tele-psychotherapeutischen Arbeit mit Kindern werden jedoch nicht entsprechend herausgearbeitet.

Im Sommer 2020, als es zu immer mehr Lockerungen im Umgang mit dem Virus kam, wurden vielfach gewohnte Settings in Präsenz wieder etabliert (Hoffnung et al. 2021). Das war für Kinder- und Jugendliche wichtig, die längere Phasen der Schulschließung bzw. des geteilten Unterrichts hinter sich hatten. Im Spätherbst 2021 traf Österreich eine zweite Covid-Welle und von 17. November bis 6. Dezember 2020 sowie von 26. Dezember bis 7. Februar 2021 wurden ein zweiter und dritter Lockdown verhängt. Damit einher gingen wieder Distance Learning für Oberstufen und geteilter Unterricht für Unterstufen. Jugendliche in beruflicher Ausbildung wurden teilweise in Kurzarbeit geschickt oder angehalten, im Home-office zu sein.

## Methodik

### Forschungsdesign

Die vorliegende explorative Studie wurde von Mitgliedern der Österreichischen Gesellschaft für angewandte Tiefenpsychologie und allgemeine Psychotherapie (ÖGATAP) initiiert und hat sich das Ziel gesetzt, Erfahrungen von SKJ-Psychotherapeut*innen aus den in der ÖGATAP beheimateten Verfahren Katathym Imaginative Psychotherapie, Hypnosepsychotherapie und Autogene Psychotherapie, sowie verwandten psychodynamischen Methoden in der psychotherapeutischen Arbeit zu erheben und eventuelle Veränderungen, die sich im Lauf der verschiedenen Phasen der Pandemie ergaben, darzustellen.

Im März 2021 wurden alle Mitglieder der ÖGATAP (*N* = 764), sowie Psychotherapeut*innen, die an der SKJ-Fortbildung der ÖGATAP teilgenommen hatten (*N* = 40), via Email eingeladen, sich an einer Online-Umfrage zu beteiligen. Die Umfrage war von Mitte März bis Mitte Mai 2021 geöffnet.

Der Fragebogen enthielt insgesamt 41 Fragen. Neben soziodemographischen Informationen wurden erlebte Veränderungen der therapeutischen Arbeit für folgende Zeiträume retrospektiv erfragt: T0 (= vor dem 1. Lockdown), T1 (= 1. Lockdown), T2 (= Lockerungen), T3 (= 2. Lockdown) und T4 (= 3. Lockdown). Pro Zeitraum wurde erfasst, wie viele Säuglingspsychotherapien, Kleinkindpsychotherapien (ca. 2–6 Jahre), Kinderpsychotherapien (ca. 6–12 Jahre) und Jugendlichenpsychotherapien (ca. 12–18 Jahre) stattfanden und in welchem Therapiemodus, wie viele und welche Psychotherapien jeweils weitergeführt werden konnten, abgebrochen wurden und neu hinzukamen. Offene Fragen fokussierten auf wahrgenommene inhaltliche und methodische Veränderungen, Veränderungen bei den Symptomen und Veränderungen in der Befindlichkeit der Psychotherapeut*innen.

### Datenauswertung

Die quantitativen Daten wurden deskriptiv ausgewertet. Die qualitativen Daten wurden mithilfe der qualitativen Inhaltsanalyse (Mayring 2019) ausgewertet. Der Ablauf der Auswertung folgte dem Prozessmodell induktiver Kategorienbildung. Nach einer ersten Durchsicht der Daten wurden nah am Datenmaterial Kategorien entwickelt und in einem zweiten Schritt thematisch geordnet. Die Codierung und anschließende Strukturierung der Kategorien erfolgten stets im Team von zumindest zwei Autor*innen, wobei ein*e Coder*in codierte und ein*e zweite*r Coder*in die Zuordnung der Textstellen überprüfte. Unstimmigkeiten wurden im Team diskutiert, ebenso wie der erste Entwurf des Kategoriensystems und seine finale Struktur.

## Ergebnisse

### Samplebeschreibung

Insgesamt nahmen 106 Psychotherapeut*innen an der Umfrage teil (Rücklaufquote 13,18 %); 23 Fragebögen wurden vollständig ausgefüllt, die restlichen Teilnehmer*innen brachen den Fragebogen ab. Die hohe Abbruchrate ist vermutlich der Länge des Fragebogens sowie den retrospektiven Fragen geschuldet, die eine nachträgliche Rekonstruktion von Patient*innenzahlen erforderten. Es wurden nur die vollständig ausgefüllten Fragebögen einer Auswertung zugeführt. Es nahmen 83 % weibliche und 17 % männliche Befragte zwischen 30 und 69 Jahren teil. Das entspricht einem durchschnittlichen Alter von 50,78 Jahren bei *N* = 23. Die Arbeitserfahrung der teilnehmenden Psychotherapeut*innen entsprach im Mittel 11,63 Jahre. 88 % arbeiten mit der Katathym Imaginativen Psychotherapie, 8 % mit Hypnosepsychotherapie und 4 % sind psychoanalytisch orientierte Psychotherapeut*innen.

### Quantitative Ergebnisse

#### Modus der Psychotherapie

(Abb. 1)

**Figure 1:**
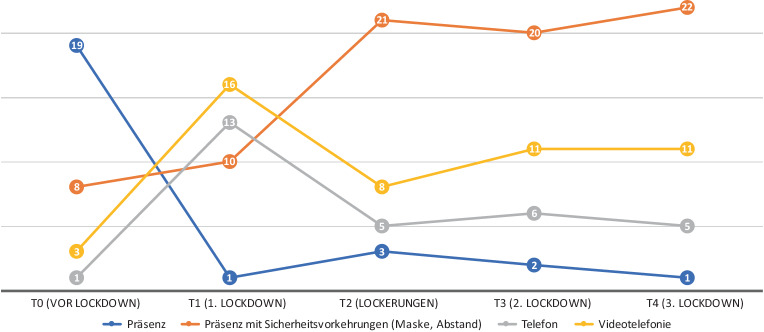

Vor dem 1. Lockdown arbeiteten die meisten Psychotherapeut*innen in Präsenz. Während des 1. Lockdowns kam es zu einem massiven Absinken der Psychotherapeut*innen, die im Präsenzmodus arbeiteten und einem Anstieg derjenigen, die Videotelefonie und Telefon nutzten. Ab T2, als die Lockerungen in Kraft traten, und bis zum Ende des Untersuchungszeitraumes arbeiteten die meisten Psychotherapeut*innen wieder im Präsenzmodus, allerdings mit Sicherheitsvorkehrungen (Maske, Abstand).

#### Patient*innenzahl

(Abb. 2)

**Figure 2:**
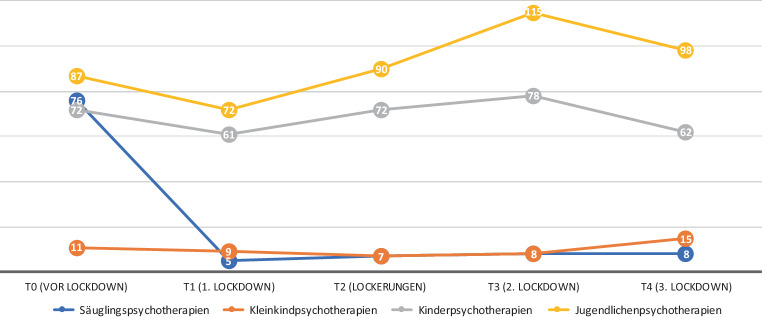

Ebenfalls änderte sich die Anzahl der Patient*innen in den verschiedenen Altersgruppen. Vor dem Lockdown (= T0) fanden die meisten Psychotherapien mit Jugendlichen statt, gefolgt von Säuglingspsychotherapien und Kinderpsychotherapien. Zu T1 (= 1. Lockdown) sank die Arbeit mit Säuglingen stark ab. Zusammen mit Kleinkindpsychotherapien blieben sie während des ganzen Untersuchungszeitraums auf einem sehr niedrigen Niveau. Psychotherapeut*innen wechselten in dieser Zeit manchmal zu Videotelefonie mit Eltern und fallweise auch zum Spielen über Videoformate. Auch Kinder- und Jugendlichenpsychotherapien sanken zu T1 ab, aber nicht so stark. Sehr deutlich zeigten die Angaben der Psychotherapeut*innen eine Zunahme an Psychotherapien mit Kindern und Jugendlichen nach T1. Zudem führten 80 % der Therapeut*innen eine Zunahme an Anfragen an, 36 % davon beschrieben diese Zunahme im Freitextfeld als *„erheblich“, „immens“, „massiv“, „stark erhöht!!!“*. In den Antworten finden sich Hinweise darauf, dass sich dieser Anstieg besonders bei Jugendlichen und im fortgeschrittenen Pandemieverlauf zeigte.

### Qualitative Ergebnisse

**Figure 3:**
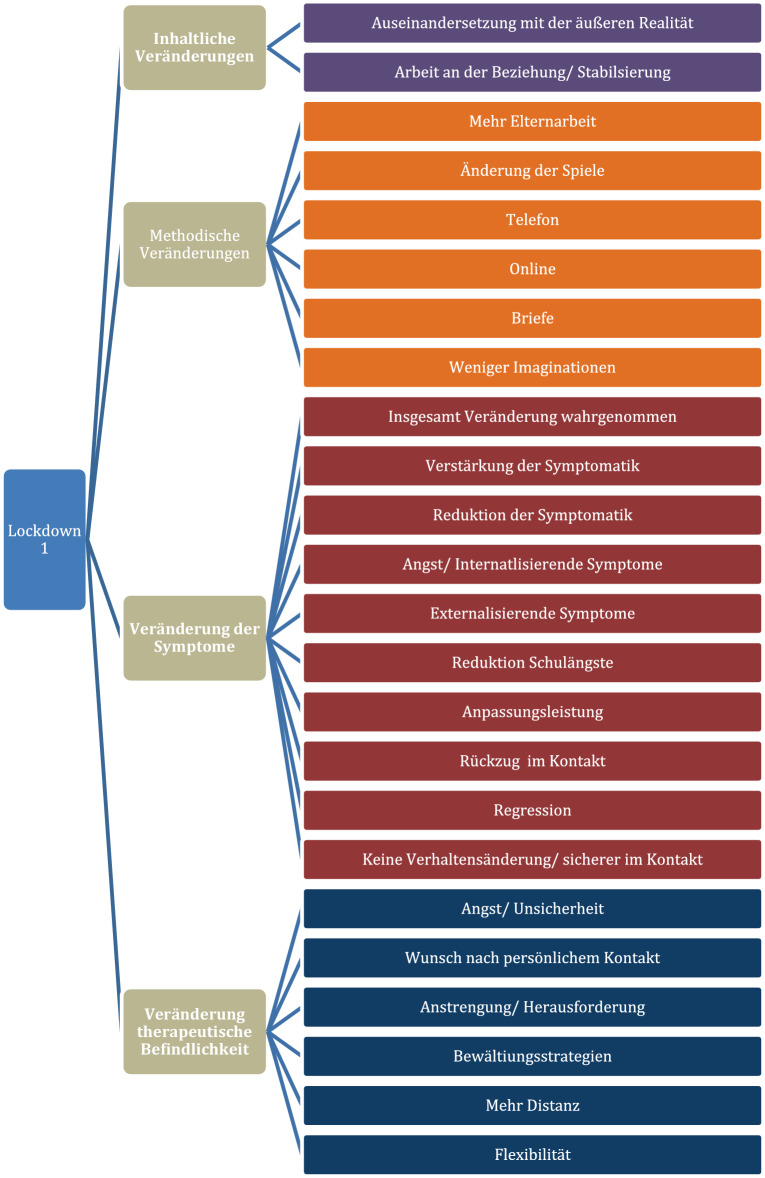

Im Folgenden werden die Ergebnisse der inhaltsanalytischen Auswertung dargestellt. Die Kapitelüberschriften entsprechen dabei den Hauptkategorien. Diesen Hauptkategorien wurden für die jeweiligen Zeitpunkte Unterkategorien zugeordnet, wie exemplarisch für den Zeitpunkt T1 in Abb. 3 dargestellt wird.

Der besseren Übersicht halber werden die Veränderungen nun anhand der Hauptkategorien über den gesamten Zeitverlauf zusammengefasst und beschrieben.

#### Inhaltliche Veränderungen

Fast alle Psychotherapeut*innen nahmen inhaltliche Veränderungen der therapeutischen Arbeit wahr. Die Auseinandersetzung mit der äußeren Realität (*„social distancing“, „Maskentragen“, „homeschooling“*) und der Pandemie rückte in den Fokus. Corona wurde als „*bestimmendes Thema*“ erlebt. Zusätzlich war die therapeutische Beziehung zentrales Thema: während zu Beginn der Pandemie die „*Aufrechterhaltung der Verbindung*“ im Mittelpunkt stand, ging es in der Zeit nach dem Lockdown (T2) häufig um den Wechsel ins ursprüngliche Setting und damit einhergehende Gefühle von Erleichterung. Die „*ursprünglichen Themen*“ schienen zumindest teilweise wieder aufgegriffen worden zu sein. Während des 2. Lockdowns (T3) beobachteten Befragte, dass das Thema Corona von manchen Patient*innen nun „ernster“ genommen wurde und der *„Abenteuer-Charakter verloren ging“*. Neues Thema speziell bei Jugendlichen waren in dieser Zeit die „*mangelnden Perspektiven und Lebensentwürfe*“.

#### Methodische Veränderungen

Nennungen zu methodischen Veränderungen bezogen sich großteils auf Setting und Rahmen. Während des ersten Lockdowns (T1) wurde vor allem der Umstieg auf Online-Psychotherapie, telefonische Psychotherapiestunden oder Kontaktaufnahmen per Brief thematisiert. Als methodische Konsequenz des Settingwechsels führten viele Befragte das Aussetzen bzw. Verringern von Imaginationen in der KIP an. Außerdem wurde beschrieben, dass sich durch das veränderte Setting das gemeinsame Spielen veränderte und kreative Lösungen gesucht werden mussten. Den neuen inhaltlichen Themen und Rahmenbedingungen entsprechend, wurde methodisch häufig von einem stabilisierenden Zugang gesprochen, einem *„haltenden Angebot“*, bzw. einem „*Fokus auf Krisenintervention*“. Mehrfach thematisiert wurden, bedingt durch den Modus Telefon oder Online, Veränderungen in der Gesprächsführung, beispielsweise eine aktivere Haltung. Zusätzlich wurde eine verstärkte oder veränderte Elternarbeit genannt. Es habe Psychotherapien gegeben, in denen nur mit den Eltern gearbeitet wurde. Es wurden vermehrt Eltern-Telefonate durchgeführt und während der Online-Psychotherapiestunden waren die Eltern mit im therapeutischen Raum.

An Setting und Rahmen musste auch nach dem ersten Lockdown (T2) weitergearbeitet werden. Vielfach wurde das ursprüngliche Setting wieder aufgenommen. Eine vermehrte Arbeit am Rahmen wurde insbesondere bei Jugendlichenpsychotherapien thematisiert, in denen das Setting brüchiger erlebt und ein häufigeres Agieren der Patient*innen beschrieben wurde. Die Wiederaufnahme des Imaginierens und Zeichnens wurde erwähnt, ebenso wie eine weiterhin vermehrte Elternarbeit. Im zweiten Lockdown (T3) wurde die intensivere Elternarbeit fortgesetzt. Die Arbeit mit Imaginationen musste erneut reduziert werden. In der Rückschau auf zweiten und dritten Lockdown (T3 und T4) finden sich erstmals Angaben zu einer verstärkten Vernetzungsarbeit mit anderen Helfer*innen und Institutionen. Es finden sich Nennungen wie „*fachärztliche Konsultationen*“, „*medikamentöse Unterstützung*“ oder „*stationäre Unterbringung*“.

#### Veränderungen der Symptomatik

Die befragten Psychotherapeut*innen beschrieben sehr eindeutige und dramatische Veränderungen schon für den Zeitraum des 1. Lockdowns (T1). Bestehende Symptome hätten sich verstärkt und neue Symptome wären entstanden. Auffallend ist die häufige Nennung von Symptomen in Zusammenhang mit Angst und Unsicherheit. Vereinzelt wurde aber auch eine Reduktion der Symptomatik genannt. Diese „Verbesserung“ wurde auf eine Entlastung durch das Wegfallen des Schulbesuchs, aber auch auf eine erhöhte Verfügbarkeit elterlicher Ressourcen zurückgeführt*. „Weniger Leistungsdruck“, „Es entspannte sich (…) etwas, da der Stressfaktor Schule wegfiel“, „überraschend gute Anpassung durch elterliche Ressourcen“*. Eine Zunahme vor allem von Angstsymptomen zeigte sich auch für den Zeitraum nach dem 1. Lockdown (T2). Wieder wurden Symptome in Verbindung gebracht mit der veränderten Schulsituation. Etwa wurde genannt, dass der erneute Schulbeginn zu *„verstärktem Druck“* geführt habe oder auch gegenteilig, dass die verkleinerten Klassen entlastend gewirkt hätten.

Die Angaben zum zweiten Lockdown (T3) weichen dann aber deutlich von bisherigen Beobachtungen ab. Psychotherapeut*innen erinnerten für diesen Zeitraum eine massive Zunahme der depressiven Symptome (inklusive expliziter Nennungen von Suizidalität), während die Angstsymptome und externalisierende Symptome nur mehr eine untergeordnete Rolle gespielt hätten. Weiterhin wurden mögliche Zusammenhänge zwischen Symptomen und der Schulsituation benannt, wie dass *„Distancelearning ein Abrutschen in eine virtuelle Welt“ *verursachen würde oder* „Schlaf-Wach-Rhythmen verschoben“* würden. Eine weitere Intensivierung depressiver Symptome (inklusive Suizidalität und Rückzug) wurde für den dritten Lockdown (T4) beschrieben.

#### Veränderungen in der Befindlichkeit der Psychotherapeut*innen

Die Pandemie und ihre Folgen für Psychotherapien führten bei vielen Psychotherapeut*innen zu Verunsicherung und Belastungen. Vereinzelt gaben die Befragten an, in Erschöpfung geraten zu sein. In den Antworten wurde deutlich, dass ein Grund dafür im Mangel an hilfreichen Angeboten von Institutionen lag. Genannt wurden etwa *„mangelnde Kassenplätze“, „restriktives Verhalten der Krankenkassen bei Bewilligungen“, „Triage auf der KJ-Psychiatrie“, „Wartezeiten“, „wenig Halt durch stationäre Einrichtungen“, „Arbeitsüberlastung, Auslaugung“. *Aber auch Rahmenbedingungen und Inhalte der psychotherapeutischen Arbeit wurden durch die Pandemie belastender. So gab es *„viel Frust zu containen, der die eigene Kapazität an die Grenzen bringt“*. Die gesteigerten Bedürfnisse und Verunsicherungen von Eltern, die vermehrt Raum in den kinder- und jugendpsychotherapeutischen Settings einnahmen, wurden mehrfach als Belastungsfaktor genannt. Zusätzlich erhöhte die deutliche Zunahme der Anfragen für SKJ-Psychotherapien das subjektive Belastungserleben der Psychotherapeut*innen. Corona-bedingte Beschränkungen reduzierten Möglichkeiten, sich als Psychotherapeut*in zu erholen. Als hilfreich im Umgang mit erhöhten Belastungsfaktoren gaben die meisten Befragten intersubjektiven Austausch an. Genannt wurden z. B. Supervision, Intervision, der Austausch im Team und ein „*gutes Netzwerk*“. Auch die eigene Offenheit und Flexibilität wurde als wichtige Ressource im Umgang mit der erschwerten Situation erlebt.

## Diskussion

Die Folgen der Pandemie für die psychische Gesundheit von Kindern und Jugendlichen sind gravierend. Psychosoziale Angebote haben versucht, dem gestiegenen Bedarf nach niederschwelliger psychosozialer Unterstützung Rechnung zu tragen und Angebote zu schaffen, die unter Pandemiebedingungen aufrechterhalten werden können (Jesser et al. 2021). Die Situation der psychotherapeutischen Versorgung von Säuglingen, Kindern und Jugendlichen und die Erfahrungen der SKJ-Psychotherapeut*innen in der psychotherapeutischen Arbeit während der Pandemie sind bislang jedoch weitgehend unbeleuchtet und daher Thema der vorliegenden explorativen Studie.

Die Ergebnisse zeigen ein massives Absinken der Säuglings- und Kleinkindpsychotherapien. Demgegenüber gab es bei den Kinder- und Jugendlichenpsychotherapien nur ein leichtes Absinken während des ersten Lockdowns und im weiteren Untersuchungszeitraum einen Anstieg. Jugendlichenpsychotherapien waren zuletzt auf einem höheren Niveau als vor der Pandemie.

Es ist anzunehmen, dass die teilweise Unmöglichkeit der Arbeit in Präsenz die Psychotherapien mit Säuglingen und Kleinkindern erschwert hat. Ebenfalls ist zu vermuten, dass es für diese Altersgruppe befremdlich sein kann, mit Sicherheitsvorkehrungen zu arbeiten (in den Antworten der Therapeut*innen auf offene Fragen gab es dazu keine vertiefenden Informationen). Bei beiden Patient*innengruppen sind die Beobachtung der Eltern-Kind Interaktion und das Anleiten in Präsenz wichtige Bestandteile der Psychotherapie. Kindertherapien bauen auf Kontakt und gemeinsamem Spiel auf. Auch das Zurverfügungstellen des therapeutischen Raumes ist für das Gelingen der therapeutischen Arbeit notwendig. Diese Bedingungen wurden durch den Lockdown im März 2020 extrem erschwert. Einige Psychotherapeut*innen wechselten im Laufe der Wochen zu Telefongesprächen mit den Eltern, um beratend zur Verfügung zu stehen. Wieder andere machten erste Erfahrungen mit Videotelefonie bei Kleinkindern, Kindern und Jugendlichen. Findige Psychotherapeut*innen entwickelten Online-Spiele, malten online oder machten Erfahrungen mit Imaginationen via Internet. In ähnlicher Weise beschreiben Cook und Zschomler (2020) die Flexibilität, mit der sich Sozialarbeiter*innen während des ersten Lockdowns in Großbritannien auf die virtuelle Arbeit mit sehr jungen Kindern einstellten. Die von den Autorinnen befragten Praktiker*innen nutzten Gesang, um die Kinder auf den Bildschirm hin zu orientieren; verwendeten virtuelle Hintergründe von Videoplattformen, um an Lebenswelten der Kinder anzudocken und bezogen das durch den Bildausschnitt sichtbare Umfeld des Kindes ein, um aus der Situation heraus Spiele zu initiieren. Trotz kreativer Ansätze und Lösungen weist das drastische Absinken der Psychotherapien bei den jüngsten Patient*innen auf die besonderen Herausforderungen hin, die sich in der Arbeit mit diesen Zielgruppen ergeben und die ein Präsenzsetting auf längere Dauer unersetzbar machen. Angesichts der wenigen Psychotherapien, die im ersten Pandemiejahr angeboten wurden, stellt sich zudem die Frage, wie es um das Befinden dieser Patient*innengruppen bestellt ist. Es könnte sein, dass Säuglinge, Kleinkinder und ihre Eltern unter den Einschränkungen der Pandemie litten und immer noch leiden und es kein entsprechendes Versorgungsangebot gibt.

Ältere Kinder und Jugendliche scheinen, möglicherweise aufgrund der bereits bestehenden Erfahrungen mit sozialen Medien, besser mit veränderten Settings zurechtgekommen zu sein und waren in der Lage, gut auf Telefonie und Videotelefonie umzusteigen. Das deckt sich mit vorhandenen empirischen Befunden (Huscsava et al. 2020). Wo live mit Maske gearbeitet wurde, waren die älteren Kinder das bereits (z. B. aus der Schule) gewohnt und konnten Befindlichkeiten dazu gut artikulieren.

Der sichere Rahmen konnte allerdings bei allen Patient*innengruppen nicht immer gehalten werden. Immer wieder berichteten Psychotherapeut*innen von Geschwisterkindern oder Eltern, die „ins Bild“ kamen. Ob Personen, die nicht sichtbar waren, ebenfalls „mit dabei“ waren, war auch nicht immer klar. Derartige Beobachtungen sind auch aus anderen Untersuchungen bekannt – selbst in Erwachsenenpsychotherapien haben Patient*innen nicht immer die Möglichkeit, sich für die Psychotherapie einen geschützten Raum in den eigenen vier Wänden zu schaffen (Jesser et al. 2022b). Diese Einschränkung muss sehr kritisch gesehen werden. Der sichere Rahmen eines therapeutischen Raumes kann in vielen Fällen und insbesondere in der Arbeit mit Kindern und Jugendlichen nur vor Ort und in Präsenz gewährleistet werden.

Hinweise auf eine verstärkte Eltern- und Vernetzungsarbeit mit Institutionen mit fortschreitendem Pandemieverlauf korrespondieren mit den von den Therapeut*innen beobachteten Veränderungen der Symptome, die mit Andauern der Pandemie stärker und krisenhafter erlebt wurden. Die wahrgenommene Zunahme vor allem depressiver Symptome zeigt sich auch quantitativ in Untersuchungen zur psychischen Gesundheit österreichischer Schüler*innen (Dale et al. 2022). Interessant ist an dieser Stelle, dass auch bei der Befindlichkeit der Psychotherapeut*innen Angst und Unsicherheit abgelöst wurden von Empfindungen wie Erschöpfung, Resignation und Verzweiflung, die klassischerweise dem depressiven Spektrum zugeordnet werden. Untersuchungen zu den Auswirkungen der Pandemie auf Wohlbefinden und psychische Gesundheit von Psychotherapeut*innen gibt es bislang keine. Weitere Forschungen wären zur Vertiefung erforderlich.

Befragte brachten Symptomveränderungen wiederholt in Verbindung mit der veränderten Schulsituation. Zunächst hätte sich durch den Lockdown für belastete Kinder und Jugendliche die Schulsituation entspannt, was sich in einer Abnahme von Schulängsten und sozialen Phobien gezeigt hätte. Diese Situation schien dann aber zu kippen, als mit dem Fortschreiten des Distance Learnings deutlich wurde, dass einigen Kindern durch die fehlende außerfamiliäre Stütze und Struktur deutliche Nachteile erwuchsen. Eine aktuelle Untersuchung der Universität für Weiterbildung Krems zeigt, dass junge Menschen an allererster Stelle schulische Sorgen anführen, wenn man sie danach fragt, was sie aktuell am meisten belastet (Jesser et al. 2022a). Auch eine Studie aus der Schweiz belegt, dass jüngere Kinder durch das Distance Learning in schulischer Hinsicht Schwierigkeiten haben und sich eine deutliche Schere auftut zwischen jenen, die es schaffen, den Anschluss zu behalten und jenen, die zurückbleiben (Tomasik et al. 2021).

Abschließend sei darauf verwiesen, dass die Ergebnisse der Studie explorativen Charakter haben. Die geringe Fallzahl und die Beschränkung auf SKJ Psychotherapeut*innen psychodynamischer Orientierung lassen keine verallgemeinerbaren Schlüsse zu; jedoch liefern die Ergebnisse wichtige Hinweise, die in zukünftigen Forschungen vertieft werden könnten. Insbesondere wäre ein Vergleich mit den Erfahrungen von Psychotherapeut*innen anderer Verfahren sowie anderer Berufsgruppen in der psychosozialen Versorgung von Säuglingen, Kindern und Jugendlichen interessant.

## Conclusio

Psychotherapeut*innen haben eine wesentliche Rolle in der psycho-sozialen Versorgung von Kindern und Jugendlichen. Im Verlauf der Pandemie kam es zu einer deutlichen Zunahme von Anfragen; auch die Eltern „drängten“ mit ihren Bedürfnissen in die psychotherapeutischen Settings. Vielen Psychotherapeut*innen gelang es, auf die veränderten Rahmenbedingungen und die erhöhten Anforderungen mit Flexibilität und kreativen Ansätzen zu reagieren. Immer häufiger wurde bei dieser Aufgabe allerdings Unterstützung in Form institutioneller Angebote, etwa kinder- und jugendpsychiatrischer Versorgung, benötigt und von Seiten der Psychotherapeut*innen beklagt, dass diese Unterstützung oft nur unzureichend oder gar nicht verfügbar sei. Es gibt Hinweise auf lange Wartezeiten für „Akutfälle“, eine Überlastung des Systems und Patient*innen die von Stelle zu Stelle wandern, ohne einen Platz zu finden. Diese Themen werden aktuell zunehmend auch von den Medien und der Öffentlichkeit wahrgenommen (Paulitsch 2022; Springer 2022). Die befragten Psychotherapeut*innen scheinen ihre Rolle in der Versorgung psychisch erkrankter Kinder und Jugendlicher während der Pandemie trotz der eigenen Betroffenheit engagiert und mit hohem Einsatz einzunehmen. Im Verlauf zeigt sich auf Seiten der Psychotherapeut*innen aber zunehmend Erschöpfung und Frustration über die strukturellen Mängel dieser Versorgung.

Dazu kommt, dass SKJ-Psychotherapeut*innen mit einer Patient*innengruppe arbeiten, deren innerpsychische Entwicklung stark von äußeren familiären und gesellschaftlichen Entwicklungen abhängig ist. In Zeiten von allgemeiner gesellschaftlicher Verunsicherung und in einer Situation, in der aus der Perspektive von Kindern und Jugendlichen Zukunftsängste ihre reale Berechtigung haben, ist anzunehmen, dass diese Ängste ihren Niederschlag in den Therapien finden und damit auch die innere Situation der Psychotherapeut*innen beeinflussen. Die weiterhin etwa durch die Klimakrise und den Ukraine-Krieg gesamtgesellschaftlich krisenhafte Entwicklung und damit die Erschwernis, positive Zukunftsperspektiven zu entwickeln, lässt vermuten, dass Kinder und Jugendliche auch in Zukunft eine Patient*innengruppe sein werden, die erhöhten Bedarf an (psychotherapeutischer) Unterstützung hat. Es ist auch anzunehmen, dass die mit der Pandemie einhergegangene Radikalisierung der Gesellschaft ebenfalls ihren Einfluss auf die innere Situation von Kindern und Jugendlichen nimmt – auch in diesem Zusammenhang wäre es interessant zu erforschen, inwieweit diese polarisierende Stimmung die SKJ-Psychotherapien beeinflusst.

Um die Patient*innengruppe der Kinder und Jugendlichen ausreichend gut zu unterstützen, bräuchte es sowohl die Erweiterung von niederschwelligen Angeboten, als auch eine deutliche Aufstockung des Kontingents für Langzeitpsychotherapien. Der von Seiten der Psychotherapeut*innen wahrgenommene Bedarf an verstärkter Eltern- und Vernetzungsarbeit gerade in Krisenzeiten führt schließlich auch zur Folgerung, dass in SKJ-Psychotherapien, anders als in Psychotherapien mit erwachsenen Patient*innen, Vernetzungsarbeit ein wesentlicher Bestandteil der therapeutischen Arbeit ist und entsprechend abgegolten werden muss.
